# Haemophilus influenzae induces steroid-resistant inflammatory responses in COPD

**DOI:** 10.1186/s12890-015-0155-3

**Published:** 2015-12-07

**Authors:** Borja G. Cosío, Andreas Jahn, Amanda Iglesias, Hanaa Shafiek, Xavier Busquets, Alvar Agustí

**Affiliations:** Hospital Universitario Son Espases-IdISPa, Palma de Mallorca, Spain; CIBER Enfermedades Respiratorias, Madrid, Spain; University of Alexandria, Alexandria, Egypt; IUNICs, Universidad de las Islas Baleares, Palma de Mallorca, Spain; Institut del tórax, Hospital Clinic, Barcelona, Spain; Department of Respiratory Medicine, Hospital Universitario Son Espases, Carretera de Valldemossa 79, 07010 Palma de Mallorca, Spain

**Keywords:** COPD exacerbation, Glucocorticoids, Colonization, Histone deacetylase, Nuclear Factor-κB, Theophylline

## Abstract

**Background:**

Chronic obstructive pulmonary disease (COPD) is an inflammatory disorder partially resistant to glucocorticoids. A reduced histone deacetylase (HDAC) activity has been proposed to explain this resistance. *Haemophilus influenzae* frequently colonizes the airways of COPD patients, where it enhances inflammation. The effects of *Haemophilus influenzae* on HDAC activity have not been investigated before.

**Methods:**

The effects of the presence or absence of *Haemophilus influenzae* ex-vivo and in vitro were studied. To this end, we determined: (1) cytokine release in alveolar macrophages (AM) from 7 patients with COPD, 5 healthy smokers, 6 healthy non-smokers and (2) HDAC activity, nuclear factor kappa B (NF-κB) activation in a macrophage-like cell line (PMA-transformed U937 cells) co-cultured with epithelial cells. Experiments were repeated with dexamethasone (1 μM) and/or the HDAC enhancer theophylline (10 μM).

**Results:**

*Haemophilus influenzae* induced a steroid-resistant inflammatory response in AM from COPD and controls and decreased HDAC activity, activated NF-κB and induced the secretion of several cytokines (IL-6, IL-8, IL-1β, IL-10 and TNF-α) (*p* < 0.001 for all comparisons) in the macrophage-like cell line. Dexamethasone reduced NF-κB activation but it did not modify HDAC activity. The addition of theophylline to dexamethasone increased HDAC activity and suppressed cytokine release completely, without modifying NF-κB activation.

**Conclusions:**

These results indicate that *Haemophilus influenzae* reduces HDAC activity and induces a NF-κB mediated inflammatory response that is only partially suppressed by glucocorticoids irrespective of having COPD. Yet, the latter can be fully restored by targeting HDAC activity.

**Electronic supplementary material:**

The online version of this article (doi:10.1186/s12890-015-0155-3) contains supplementary material, which is available to authorized users.

## Background

Chronic Obstructive Pulmonary Disease (COPD) is characterized by an abnormal inflammatory response of the lungs to, mostly, cigarette smoke [1]. This response is considered abnormal because, it is enhanced with respect to that occurring in smokers with normal lung function, it progresses with disease severity [2], it persists despite smoking cessation [3, 4] and it is relatively resistant to the anti-inflammatory effects of steroids [5]. The mechanisms underlying these abnormalities are unclear, but their better understanding may facilitate the development of novel and more effective therapeutic strategies for these patients.

Nuclear factor kappa B (NF-κB) is a transcription factor that regulates the expression of multiple immune and inflammatory genes [6]. However, the augmented inflammatory response that occurs in severe COPD is not associated with more NF-κB activation but with decreased total histone deacetylase (HDAC) activity [7]. HDAC and histone acetyl-transferases (HAT) are families of enzymes that regulate chromatin structure, thus inflammatory gene expression [8]. Acetylation of histones by co-activator proteins, such as CREB-binding protein (CBP), p300 and TAF_II_250, unwind DNA and allow transcription factors and RNA polymerase II to switch gene transcription on [9]. Conversely, de-acetylation of core histones is associated with transcriptional repression [10]. It has been previously shown that glucocorticoid suppression of inflammatory genes requires recruitment of HDACs to the activation complex by ligand-activated glucocorticoid receptor (GR) [11], and reduced HDAC activity has been found to be related to glucocorticoid insensitiveness in COPD [5]. Interestingly, theophylline enhances HDAC activity in stable COPD patients and reverts steroid insensitivity *in vitro* and *ex vivo* [12].

Chronic bacterial colonization of the airways contribute to the inflammatory response of COPD [13] and to disease progression [14–16]. Airway infection is also a key pathogenic mechanism of COPD exacerbations [13, 15, 17–19] which, are characterized by a flare-up of airway inflammation [20–22]. *Non-typeable Haemophilus influenzae* (NTHi) is frequently isolated from COPD patients both when clinically stable and during exacerbations [17, 19]. NTHi induces a strong inflammatory response in the respiratory tract with activation of immune responses. NTHi acts primarily through the NF-*κ*B signaling pathway to increase inflammation [23]. This effect may be enhanced by the effect of co-secreted cytokines from epithelial cells, such as by the addition of TNF-*α* and the recruitment of neutrophils [23, 24]. Alveolar Macrophages are also a major source of TNF-*α* and IL-1*α* production as well as professional phagocytes and antigen-presenting cells. But, smoking impairs the phagocytosis in macrophages and defense mechanisms of the bronchial epithelial cells [25, 26] which is not restored with dexamethasone treatment [25].

The potential effects of *Haemophilus influenzae* upon inflammatory responses or HDAC activity have not been investigated before. Likewise, whether or not these effects are susceptible to the action of pharmacologic effectors is unknown. This study sought to get further insight into the pro-inflammatory effects of *NTHi* and the potential for pharmacological intervention. To this end, we first determined the inflammatory response elicited by *NTHi* in cultured alveolar macrophages and, then, we investigated the effects of altering the NF-κB and HDACs activity pathways in macrophage-like cells.

## Methods

### Bacterial strains and preparation

*Haemophilus influenzae* strain 05-118741 is a nontypable isolate kindly provided by Dr A. Oliver and previously used [27]. *NTHi* was grown in chocolate agar plates or in tryptone soya broth (TSB) supplement with Fildes enrichment (sTSB) at 37 °C in 5 % CO_2_.

### Population

Seven patients with diagnosis of COPD according to international guidelines [1], 5 smoker and 6 never-smoker patients without evidence of airway obstruction on spirometry undergoing bronchoscopy for clinical reasons were included. Patients with active inflammatory or infectious diseases, receiving treatment with antibiotics, immunosuppressive agents, oral or inhaled corticosteroids or theophylline, or patients with a COPD exacerbation within the previous 6 weeks were excluded. All participants signed an informed consent approved by the local Ethics Committee. Fiberoptic bronchoscopy was performed and bronchoalveolar lavage (BAL) was obtained from the right middle lobe as previously described [12]. Alveolar macrophages were isolated from the BAL, incubated in 6-well plates and cultured at 37 °C in a humidified atmosphere with 5 % CO2 in RPMI 1640 medium containing 0.5 % fetal calf serum (FCS), 5 % HEPES and supplemented with antibiotics (50 U/ml penicillin and 50 Ag/ml streptomycin) for 24 h. The protocol was approved by the Ethic Committee of the Balearic Islands.

### Cell culture

U937 cells, a human monocyte cell line (ATCC designation CRL-1593.2), were cultured at 37 °C in a humidified atmosphere with 5 % CO_2_ in RPMI 1640 medium containing 10 % FCS, 5 % HEPES and supplemented with antibiotics (50 U/ml penicillin and 50 Ag/ml streptomycin). They were grown to 70 % confluence before incubation for 24 h in 0.5 % FCS medium. To transform U937 cells to macrophage-like cells prior to culture, cells were exposed to 100nM phorbol 12-myristate 13-acetate (PMA) for 72 h before adherent cells were scraped and incubated in RPMI 1640 medium containing 0.5 % FCS [28].

To mimic the alveoli microenvironment, A549 epithelial cells (ATCC, designation CCL-185) were co-cultured with the transformed U937 cells in a 2:1 (A549:U937) ratio. Both A549 and U937 cell lines were and grown in RPMI-1640 media supplemented with 1 % Pen-Strep, 5 % HEPES and 10 % heat-inactivated FBS. The U937 cells were plated in 24 dishes culture plate and A549 cells were plated directly on the transwell inserts (0.4 mm, BD Biosciences) in culture medium. Prior to co-culture, A549 cells and macrophages were washed with RPMI containing 0.5 % FCS (basal medium) for 24 h, then the inserts were placed in each well.

Cells were infected at MOI of 100:1 [29]. Simultaneously, cells were treated with dexamethasone (1 μM), theophylline (10 μM) and a combination of dexamethasone and theophylline, at the same concentrations, which we know are effective from previous experiments [30]. Supernatants were collected after 18 h and kept at -20 °C. In different experiments, cells were isolated after 4 h and nuclear proteins extracted using a Nuclear extract kit ® (Active Motif, CA, USA) and kept at -80 °C. Experiments were repeated 3 times by duplicate.

### Western blotting

Nuclear extracts were measured by sodium dodecylsulphate-polyacrylamide gel electrophoresis (SDS-PAGE) and Western blot analysis using enhanced chemiluminescence (ECL; Amersham, Amersham, UK). Proteins were size fractionated by SDS-PAGE and transferred to Hybond-ECL membranes. Inmunoreactive bands were detected by ECL using specific antibodies for Intercellular Adhesion Molecule 1 (ICAM 1) (#4915) obtained from Cell Signaling technology, Inc. (Danvers, MA, USA) as previously described [11].

### Cytokine determination

Culture supernatant was analyzed for cytokine determination (interleukin (IL)-10, IL-6, IL-1β, IL-8 and tumor necrosis factor (TNF)-α) with BD Cytometric Bead Array (CBA) Human inflammation Kit ® (BD Biosciences) following manufacturer’s instructions. Assay sensitivity as expressed by the manufacturer was as follows: 3.6 pg/ml for IL-8, 7.2 pg/ml for IL-1β, 2.5 pg/ml for IL-6, 3.3 pg/ml for IL-10 and 3.7 pg/ml for TNF-α.

### HDAC activity

HDAC activity of nuclear extracts was measured with a non-isotopic assay using a fluorescent derivative of epsilon-acetyl lysine (HDAC fluorescent Activity Assay Kit, BIOMOL, Plymouth, PA). This assay is based on the Fluor de Lys^TM^ (Fluorogenic Histone deAcetylase Lysyl Substrate/Developer) Substrate and Developer combination. Briefly, deacetylation of the substrate sensitizes the substrate so that, in the second step, mixing with the Color de Lys Developer causes an increase in yellow color intensity. HeLa Nuclear Extract provided by the manufacturer was used as a positive control, and as negative control it was used the potent HDAC inhibitor Trichostatin A [11]. The assay was performed exactly as recommended by the manufacturer and emitted light detected at 460 nm in a fluorometric plate reader.

### NF-κB activity

NF-κB activation was assessed in transformed-U937 cells using the TransAM NF-κB p65 Transcription Factor Assay Kit (Active Motif, CA, USA). Briefly, nuclear extracts were prepared according to the Nuclear Extract Kit (Active Motif, CA, USA) and 3ug of nuclear proteins were added to a 96- well plate containing an immobilized oligonucleotide corresponding to the NF-κB consensus site (5VGGGACTTTCC-3 V) and to which specifically binds the active form of NF-κB present in the nuclear extract. Next, an antibody against an epitope of the p65 (RelA) subunit of NF-κB was added. Incubation with the adequate peroxidase-conjugated secondary antibody and with a chemilumi-nescent reagent provided a signal that was quantified by means of the Bio-Tek FL-800 reader. Positive controls were made using a nuclear extract of Jurkat cells provided by the manufacturer and negative controls through competitive binding with a wild-type consensus oligonucleotide, also provided as a competitor for NF-κB binding. Moreover, the addition of a mutated consensus oligonucleotide did not have any effect on NF-κB binding.

### Statistical analysis

Results are expressed as means ± standard deviation of the mean (SD) of at least 3 different experiments in duplicate. All the data were tested for normal distribution by Kolmogorov - Smirnov 2-sample test. Comparison between experimental groups was performed using the Mann–Whitney U or independent t- test for not normally and normally distributed data respectively and analysis of variance (ANOVA). All statistical testing was performed by using a two-sided 5 % level of significance using GraphPad Prism software (GraphPad Software Inc., San Diego, CA) and SPSS package (version 18; Chicago, USA). Significant *p* value was considered if p ≤ 0.05.

## Results

Patient’s characteristics are summarized in Table 1. COPD patients were predominantly GOLD II (moderate), and they were receiving treatment with long-acting bronchodilators. None of them show evidence of infection or colonization in the microbiology of the bronchial aspirate after bronchoscopy.

**Table 1:** Population characteristics. Data are presented as mean ± SD unless it is otherwise stated

Characteristic	COPD	Smokers	Non-smokers
	(*n* = 7)	(*n* = 5)	(*n* = 6)
Age	55.0 ± 4.4	59.4 ± 11.6	56.8 ± 16.1
Gender (M/F)	6/1	4/1	5/1
Smoking history			
Smoking index	69.3 ± 16.8	42.5 ± 3.5	0
Current/former smoker	5/2	4/1	0
FEV_1_/FVC (%)	60.7 ± 7.9	84 ± 8	83.6 ± 3.6
FEV_1_ (L)	2.5 ± 0.4	2.9 ± 0.7	3.6 ± 1.0
FEV_1_% predicted	78.3 ± 16.0	96 ± 16.3	107.2 ± 17.5
BAL:			
Volume of BAL recovered (ml)	37.9 ± 10.7	51 ± 18.2	62 ± 20.8
Total cells (cells/μl)	167 ± 54.9	233.5 ± 116	210 ± 29.7
*Differential cell count*:			
Macrophages (cells/μl)	21.68 ± 6.46	72.39 ± 11,61	75,94 ± 2,35 22,07 ± 3.02
Lymphocytes (cells/μl)	31.93 ± 5,29	17.9 ± 7,29	
Neutrophils (cells/μl)	45,93 ± 8,59	9,69 ± 4,5	1,92 ± 0,71

**Fig. 1:**
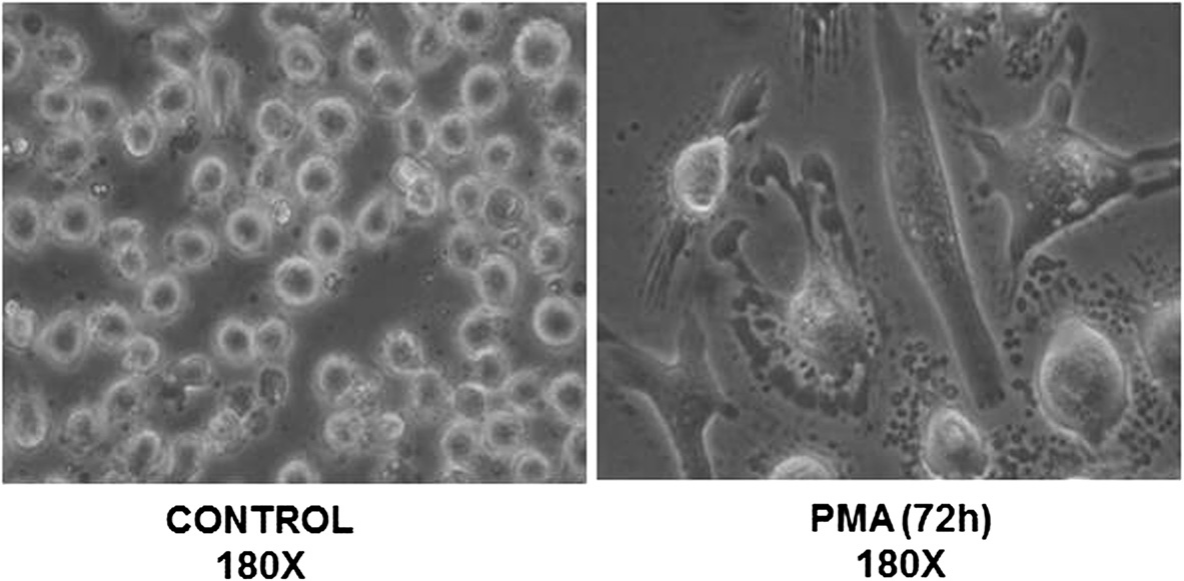
Phenotypic and morphologic changes observed in U937 cells after treatment with PMA (100 nM) in the presence of Haemophilus influenzae

U937 cells treated with PMA for 72 h showed phenotypic (ICAM-1 expression) and morphological changes characteristic of alveolar macrophages (Fig. 1). Cell viability assessed by trypan blue dye exclusion was >95 % even 12 h after infection.

### Pro-inflammatory effects of *H. influenzae*

**Fig. 2:**
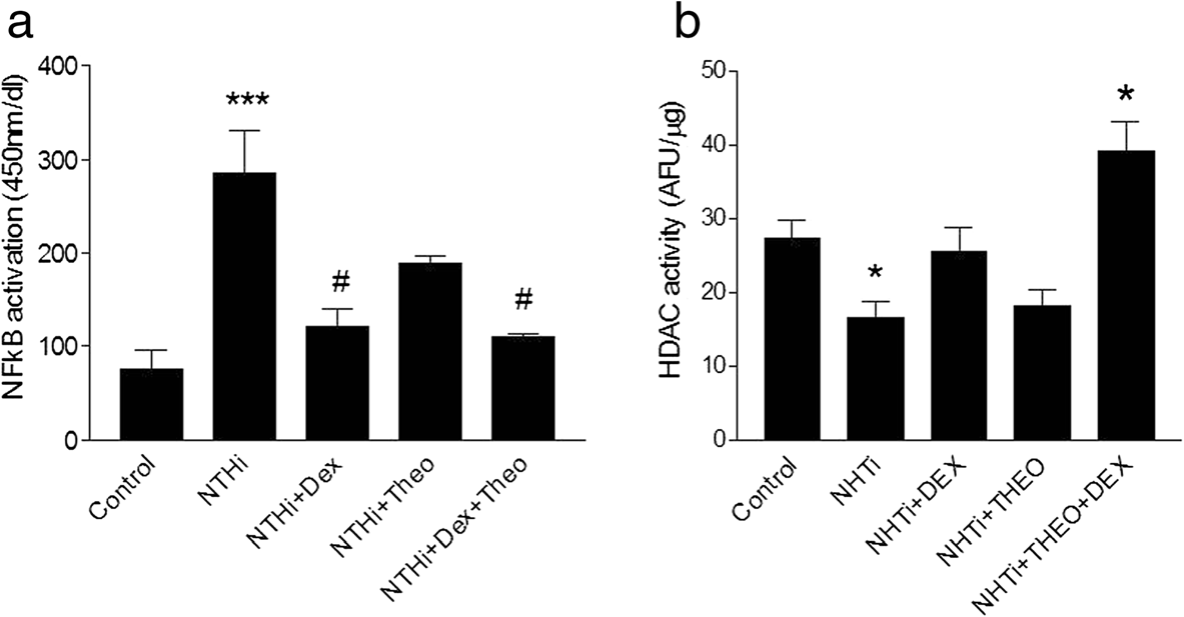
Effect of Dexamethasone and Theophylline on NF-kB activation activity in macrophage-like cells after NTHi infection (**a**). Effect of Dexamethasone and Theophylline on HDAC activity in macrophage-like cells after NTHi infection (**b**). Abbreviations Dex: dexamethasone 1 μM, Theo: theophylline 10 μM, NTHi: Nontypeable Haemophilus influenzae. (# *p* < 0.05 over stimulated cells, ***p < 0.001 over control, *p < 0.05 over control)

**Fig. 3:**
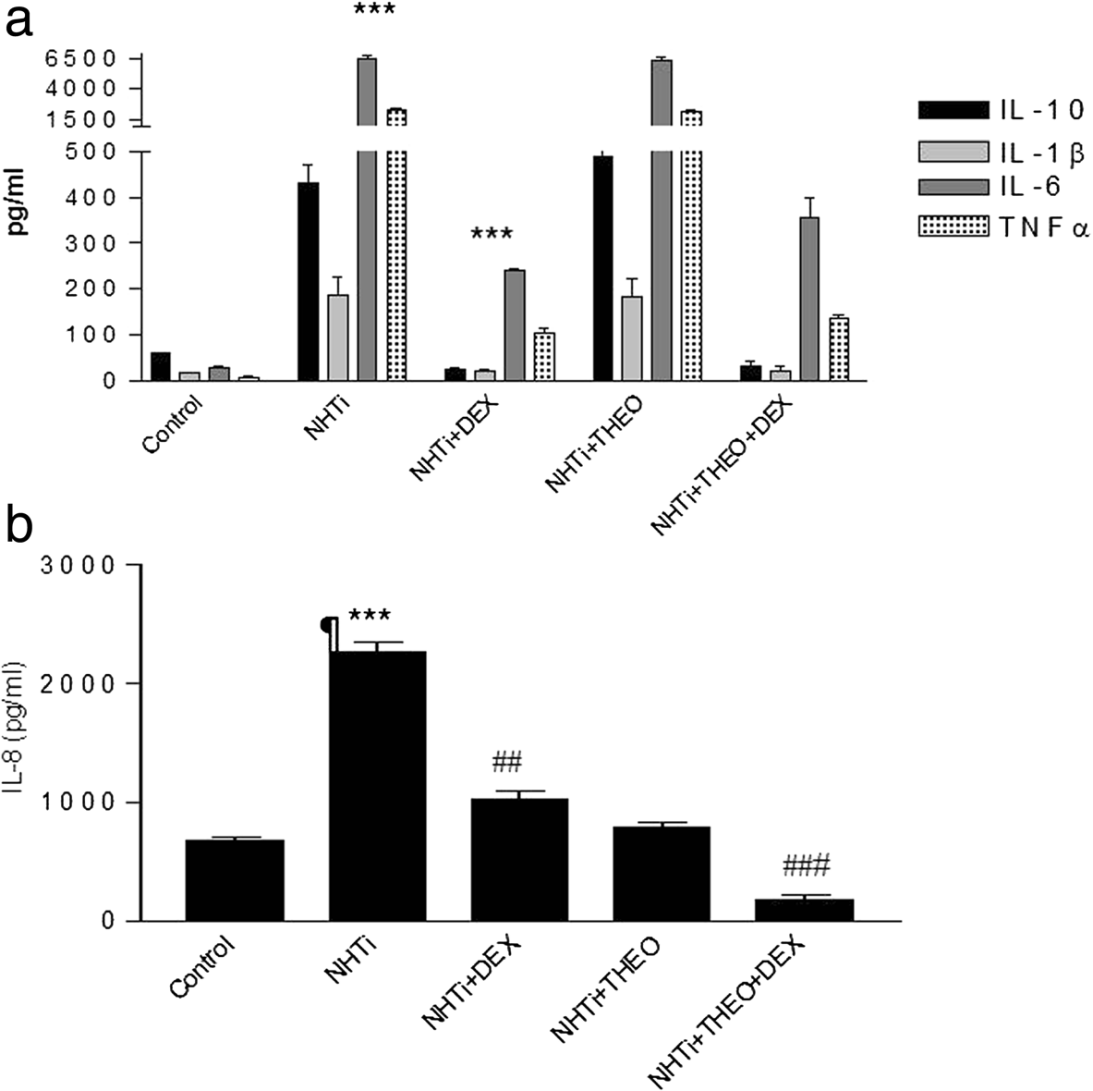
Effect of Dexamethasone and Theophylline on IL-6, IL-1 β, IL-10 and TNF-α (**a**) and IL-8 (**b**) in macrophage-like cells after *Haemophilus influenzae* infection. Abbreviations Dex: dexamethasone 1 μM, Theo: theophylline 10 μM, NTHi: Nontypeable Haemophilus influenzae. (***p < 0.001 over control, ## *p* < 0.01 over stimulated cells, ¶ *p* < 0.05 over Dex + NTHi)

*NTHi* induced an inflammatory response in COPD alveolar macrophages characterized by a statistically significant increase in all inflammatory cytokines (Additional file 1: Figure S1, online supplement). This inflammatory profile was similar to the response observed in control patients (both smokers and never-smokers) and in the cell line models. In macrophage-like cells, *NTHi* activated NF-κB (Fig. 2a) and induced a strong inflammatory reaction as shown by the significant increase in the release of IL-1β (from 17 ± 6 to 186 ± 82 pg/ml, *p* < 0.001), IL-6 (from 30 ± 6 to 6427 ± 482 pg/ml, *p* < 0.001), TNF-α (from 8 ± 4 to 2275 ± 385 pg/ml, *p* < 0.001), IL-10 (from 59 ± 5 to 429 ± 91 pg/ml, *p* < 0.001) and IL-8 (from 686 ± 50 to 2267 ± 289 pg/ml, *p* < 0.001); Fig. 3a and b. Interestingly, we also observed that HDAC activity was significantly reduced by *NTHi* infection (*p* <0.05) (Fig. 2b).

### Inflammatory suppression

**Fig. 4:**
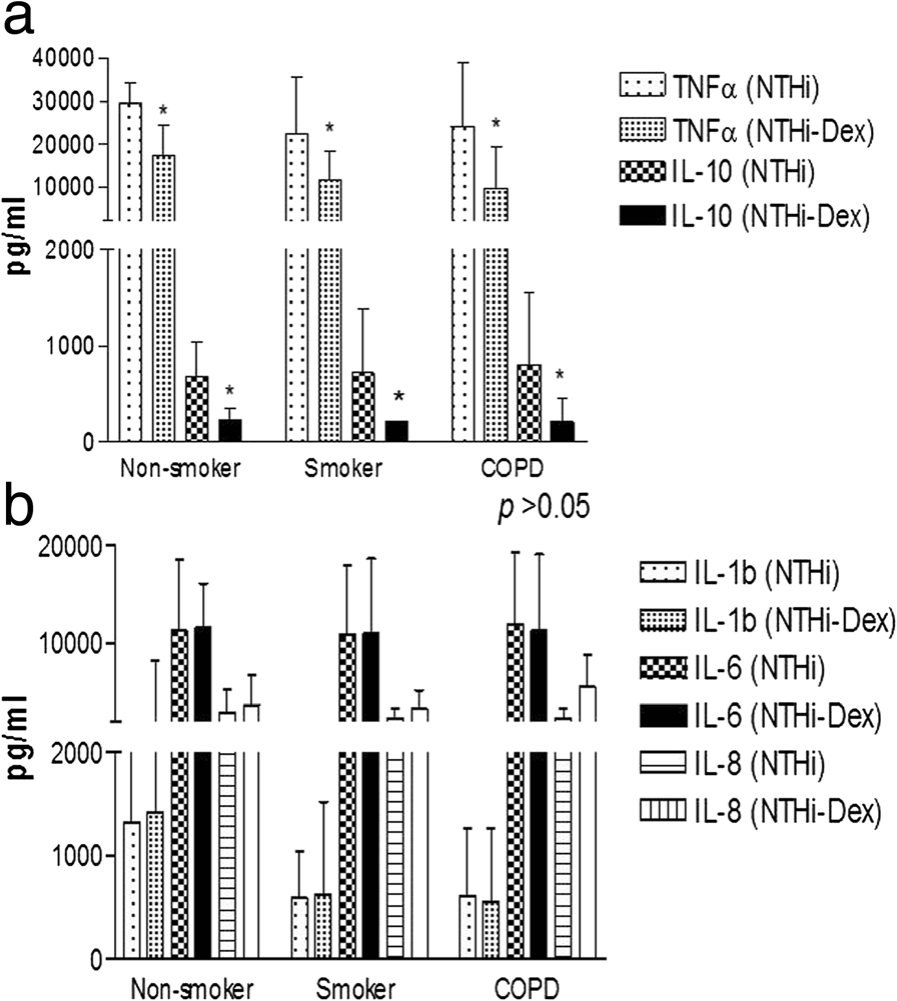
Effect of dexamethasone on TNFα and IL-10 (**a**) and IL-6, IL-8 and IL-1β (**b**) released from alveolar macrophages obtained from COPD patients, smoker and non-smoker controls Abbreviations Dex: dexamethasone 1 μM, NTHi: Nontypeable Haemophilus influenzae (*p < 0.05 over infected cells with NTHi)

The inflammatory response elicited by *NTHi* was partially suppressed by dexamethasone in COPD alveolar macrophages as well as macrophages from control smokers and healthy non-smokers (Fig. 4). Thus, TNF-α, and IL-10 release were significantly reduced by dexamethasone (with average suppression of 56 % and 68 % respectively, *p* <0.05; Fig. 4a) whereas IL-1β, IL-6 and IL-8 were not significantly suppressed in any of the *NTHi*-infected macrophages, irrespective of having COPD (p >0.05, Fig. 4b).

In the co-culture, the addition of dexamethasone in the presence of infection reduced the release of TNF-α and IL-10 by 82 %, from 13359.6 ± 385.7 to 2371.2 ± 594.4 pg/ml and 92 % from 860.3 ± 237.4 to 641.4 ± 116.5 respectively (*p* < 0.05) without significant change on IL-1β, IL-6 and IL-8 release (Additional file 1: Figure S2, online supplement).

### Effects of targeting HDAC activity

In macrophage-like cell line, the HDAC enhancer theophylline by itself produced a non-significant reduction of NF-κB activation (Fig. 2a) and failed to modify the release of any of the cytokines measured (Fig. 3a). Theophylline itself did not affect HDAC activity (Figure 2b). The combination of theophylline and dexamethasone did not modify NF-κB activation nor enhanced further reduced release of IL-6, IL-10, IL-1β and TNFα observed with steroids alone (Figs. 2a, b and 3a). Yet, this combination resulted in a significantly greater suppression of IL-8 release (92 vs 58 %) as compared with dexamethasone alone (1024 ± 150 vs. 174 ± 65 pg/ml, *p* <0.05; Fig. 3b) and significantly boosted HDAC activity (Fig. 2b).

## Discussion

This study confirms that *NTHi* activates a NF-κB-dependent inflammatory response in alveolar macrophages [23, 31], and shows for the first time that a simultaneous decrease in global HDAC activity occurs. This is relevant because it is shown here that this inflammatory response cannot be fully suppressed by the most potent anti-inflammatory drug, glucocorticoids. This partial steroid resistance does not appear to be exclusively related to COPD, as previously thought, since it is also induced in macrophages from smokers and non-smokers not suffering from COPD. Further, in our experimental cell model, the combination of dexamethasone and theophylline can restore HDAC activity and suppress IL-8 almost completely.

### Previous studies

It is well established that bacteria can stimulate the expression of many cytokines and chemokines, especially IL-8, in different cell types [32]. This up-regulation normally (but not exclusively) involves the activation of the NF-κB pathway [33]. Further in a murine model of airway infection, *NTHi* upregulated the expression of ICAM-1 in epithelial cells and increased chemokine levels and neutrophil recruitment in the airway [34]. Our results agree with these observations because we also observed NF-κB activation, a significant increase in cytokine release and enhanced expression of ICAM-1.

### Interpretation of novel findings

HDAC activity is progressively reduced as COPD severity increases [7]. This is associated with histone-4 acetylation of the IL-8 promoter and increased IL-8 messenger RNA (mRNA) [7]. The mechanisms underlying these observations are unclear. We found that *NTHi*, a bacterial pathogen often isolated from the airways of patients with moderate to severe COPD [17, 19], induces partial steroid resistance *ex vivo* and reduces HDAC activity *in vitro*, thus offering a potential explanation for the observed reduction of HDAC in patients with advanced COPD. Thus, we propose that the reduced activity of HDAC induced by *NTHi* that we observed in our study is likely to be due to post-translational modifications of HDAC (nitration or phosphorylation) [35, 36]. Other pathogens might act through the same mechanisms in COPD patients, as it has been shown that they can induce a NF-kB inflammatory response [37, 38]. Actually, in a recent and similar study, *Moraxella catarrhalis* (another bacterial pathogen frequently isolated from patients with COPD) enhanced the acetylation of histone H3 and H4, both globally and at the promoter site of the IL-8 gene, in bronchial epithelial cells [37]. Further, preventing histone deacetylation by the histone deacetylase inhibitor trichostatin A augmented the IL-8 response induced by *M. catarrhalis* [37]. The role of other bacteria or viruses related to COPD exacerbations have never been explored.

In our study *in vitro*, glucocorticoids abolished the release of most (IL-1β, IL-6, TNF-α and IL-10) but not all (IL-8) cytokines induced by *NTHi*. Yet, the latter was fully abolished when steroids were combined with theophylline, a drug that restored HDAC activity. Suppression of cytokine expression by glucocorticoids is not exclusively dependent on NF-κB inhibition, and several post-transcriptional events, such as stabilization of IL-8 mRNA [39], and the participation of other transcription factors or co-factors is required to regulate the expression of different cytokines [40]. Our data support a role for HDAC in this regulatory process since theophylline, a known HDAC activator [12, 30, 41], could restore the relative resistance to glucocorticoids of *NTHi* induced-IL-8 expression. Yet, theophylline alone did not affect IL-8 release induced by *NTHi*. This is likely due to the fact that enhanced HDAC activity by itself is not capable to suppress inflammatory gene transcription because it needs to be recruited to the active pro-inflammatory transcriptional complex by the GR [41]. This can explain the relative lack of effect of theophylline alone in suppressing IL-8 release induced by *NTHi*, as well as its effect when combined with dexamethasone that we observed in our study.

### Clinical implications

In patients with COPD, bacterial pathogens (frequently *NTHi*) are often found [17, 19] in their airways. This contributes significantly to disease progression via enhancement and/or persistence of the inflammatory reaction elicited by tobacco smoking [14–16]. In this context, our experimental results might have some clinical relevance. Given that COPD is characterized by relative steroid resistance [5, 42–44] and that IL-8 is a potent chemokine for neutrophil recruitment [45], these observations suggest that re-addressing the histone acetylation/deacetylation imbalance with a potent HDAC activator like theophylline may be effective in restoring glucocorticoid sensitiveness in these patients thus enhancing their therapeutic potential.

### Potential limitations

Our study has several potential limitations that deserve comment. First, the patient population is small and our results *in vitro* cannot be readily extrapolated to *in vivo* conditions. Second, we are not showing the ultimate mechanisms responsible for our observations (post-translational modifications). Although we think that our results are relevant, further mechanistic studies are needed to identify potential therapeutic targets.

## Conclusions

Our data provides novel information on the molecular mechanisms used by *NTHi* to activate the inflammatory response in macrophages. We found that it induces a decrease in HDAC activity that can be reverted by theophylline, and that this is likely to underlie the relative resistance of IL-8 expression to be suppressed by glucocorticoids. These findings may be relevant for a better understanding and treatment of COPD.

## Additional file

Additional file 1:
**Figure S1.** Effect of H. influenza infection on inflammatory cytokines release from alveolar macrophages obtained from COPD patients, smoker and non-smoker controls. Abbreviations NTHi: Nontypeable Haemophilus influenzae (**p* < 0.05 over control cells). **Figure S2.** Effect of Dexamethasone and Theophylline on cytokine release in co-culture cells after *Haemophilus influenzae* infection. Abbreviations Dex: dexamethasone 1 μM, Theo: theophylline 10 μM, NTHi: Nontypeable Haemophilus influenzae (**p* < 0.05 over infected cells with NTHi). (PDF 18 kb)

## Abbreviations

COPD: Chronic obstructive pulmonary disease
HDAC: Histone deacetylase
AM: Alveolar macrophages
NF-κB: Nuclear factor kappa B
GR: Glucocorticoid receptor
BAL: Bronchoalveolar lavage
FCS: Fetal calf serum
ICAM-1: Intercellular adhesion molecule 1
IL: Interleukin
TNF: Tumor necrosis factor
