# Supplementary figures and images for: Haemophilus influenzae induces steroid-resistant inflammatory responses in COPD
Source: BMC Pulm Med. 2015 Dec 7;15:157. doi: 10.1186/s12890-015-0155-3 (PMC4672509; doi:10.1186/s12890-015-0155-3)

Figure S1

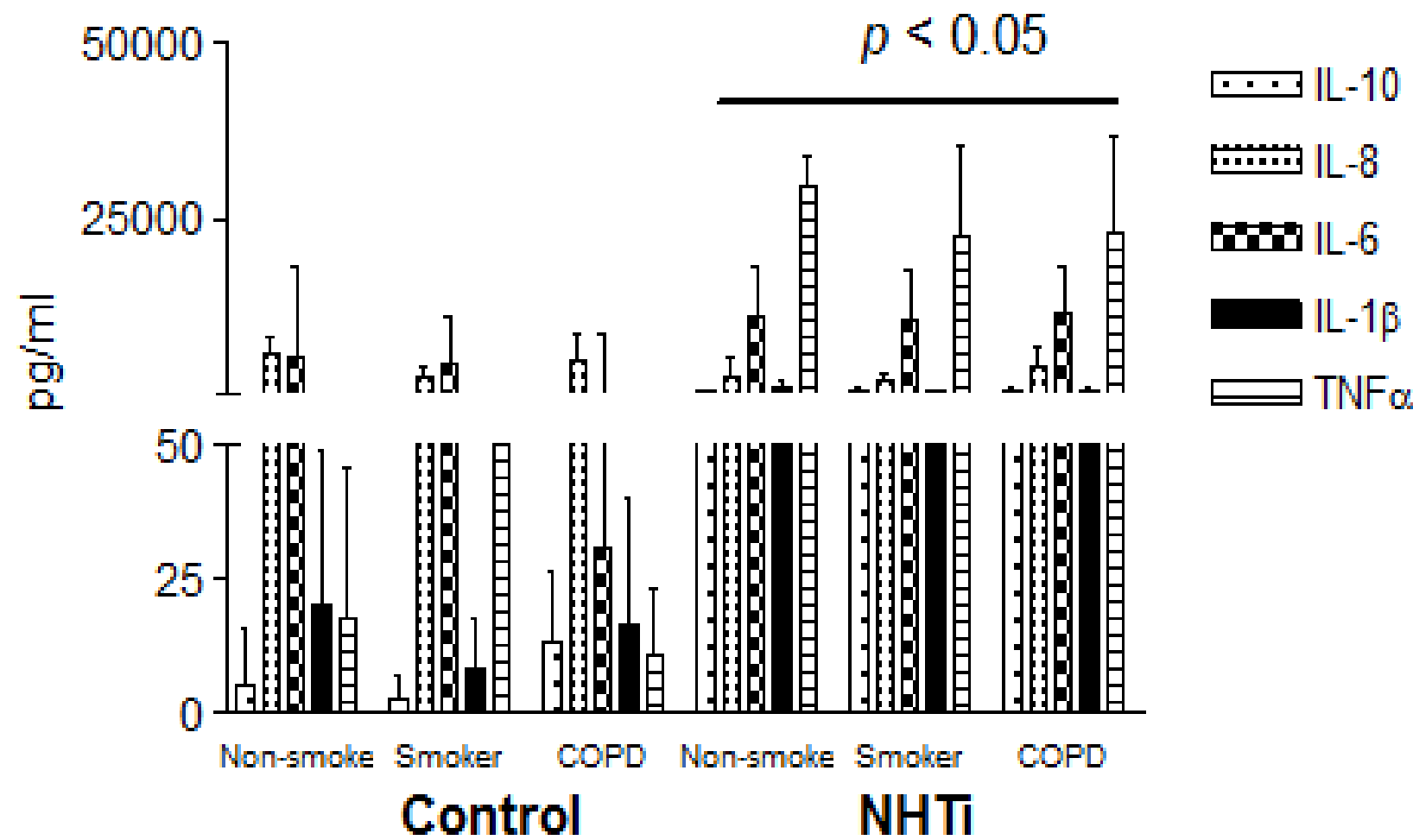

Figure S2

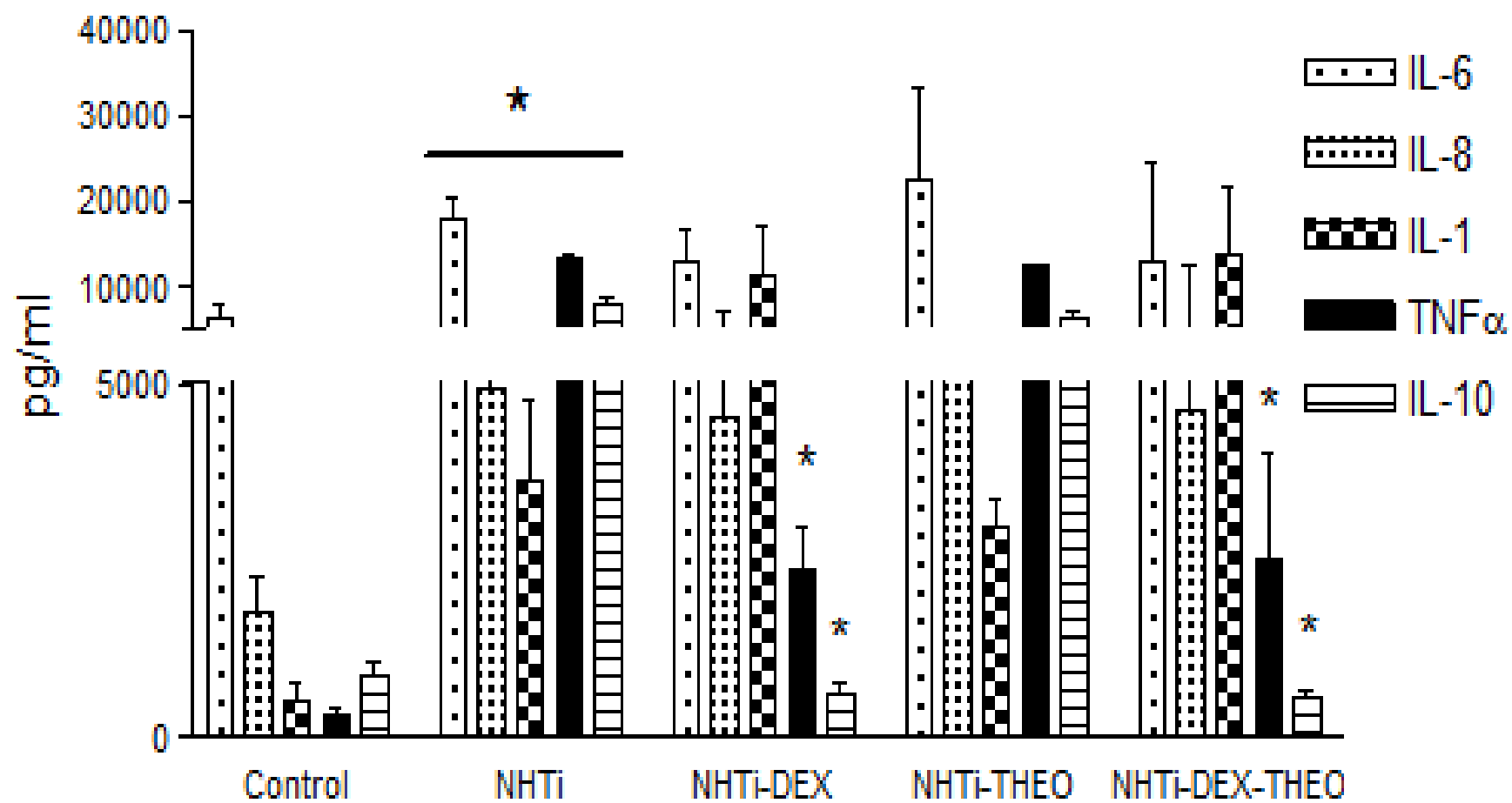

Supplement: Additional file 1: — Figure S1. Effect of H. influenza infection on inflammatory cytokines release from alveolar macrophages obtained from COPD patients, smoker and non-smoker controls. Abbreviations NTHi: Nontypeable Haemophilus influenzae (*p < 0.05 over control cells). Figure S2. Effect of Dexamethasone and Theophylline on cytokine release in co-culture cells after Haemophilus influenzae infection. Abbreviations Dex: dexamethasone 1 μM, Theo: theophylline 10 μM, NTHi: Nontypeable Haemophilus influenzae (*p < 0.05 over infected cells with NTHi). (PDF 18 kb) [file 12890_2015_155_MOESM1_ESM.pdf]
